# The Association between Non-Invasive Ventilation and the Rate of Ventilator-Associated Pneumonia

**DOI:** 10.3390/diseases11040151

**Published:** 2023-10-27

**Authors:** Hollie Saunders, Subekshya Khadka, Rabi Shrestha, Arvind Balavenkataraman, Alexander Hochwald, Colleen Ball, Scott A. Helgeson

**Affiliations:** 1 Department of Pulmonary and Critical Care, Mayo Clinic, 4500 San Pablo Road, Jacksonville, FL 32224, USA; subekshya.khadka@gmail.com (S.K.); drrabishresthasai@gmail.com (R.S.); balavenkataraman.arvind@mayo.edu (A.B.);; 2 Department of Quantitative Health Sciences, Mayo Clinic, Jacksonville, FL 32224, USA

**Keywords:** mechanical ventilation, non-invasive ventilation, ventilator-associated pneumonia

## Abstract

Ventilator-associated pneumonia (VAP) has significant effects on patient outcomes, including prolonging the duration of both mechanical ventilation and stay in the intensive care unit (ICU). The aim of this study was to assess the association between non-invasive ventilation/oxygenation (NIVO) prior to intubation and the rate of subsequent VAP. This was a multicenter retrospective cohort study of adult patients who were admitted to the medical ICU from three tertiary care academic centers in three distinct regions. NIVO was defined as continuous positive airway pressure (CPAP), bilevel positive airway pressure (BiPAP), or high-flow nasal cannula (HFNC) for any duration during the hospitalization prior to intubation. The primary outcome variable was VAP association with NIVO. A total of 17,302 patients were included. VAP developed in 2.6% of the patients (444/17,302), 2.3% (285/12,518) of patients among those who did not have NIVO, 1.6% (30/1879) of patients who had CPAP, 2.5% (17/690) of patients who had HFNC, 8.1% (16/197) of patients who had BiPAP, and 4.8% (96/2018) of patients who had a combination of NIVO types. Compared to those who did not have NIVO, VAP was more likely to develop among those who had BiPAP (adj OR 3.11, 95% CI 1.80–5.37, *p* < 0.001) or a combination of NIVO types (adj OR 1.91, 95% CI 1.49–2.44, *p* < 0.001) after adjusting for patient demographics and comorbidities. The use of BiPAP or a combination of NIVO types significantly increases the odds of developing VAP once receiving IMV.

## 1. Introduction

Ventilator-associated pneumonia (VAP) is defined as a new pneumonia developing 48 h after endotracheal intubation and mechanical ventilation. Often, this will be determined by a new or progressive lung infiltrate on imaging with clinical evidence of an infection and an isolated pathogen in microbiologic sampling [1,2]. It is estimated that around 5–40% of patients receiving invasive mechanical ventilation (IMV) develop VAP with associated mortality rates around 10–50% [1,2,3]. VAP and its associated mortality have significant effects on patient outcomes, including prolonging the duration of both IMV and intensive care unit (ICU) length of stay (LOS), as well as imposing a significant economic burden [3]. For these reasons, efforts have been made to identify and prevent factors contributing to VAP incidence by creating “bundles” to aid in VAP prevention. These bundles include specifics on patient positioning, oral care, and most importantly, minimizing the length of IMV. These are based on the hypothesis that VAP may be caused by the aspiration of gastric and oral secretions and the biofilm contamination of the endotracheal tube [4]. Despite these interventions, the incidence of VAP has been largely unchanged and remains a significant burden on both patients and the healthcare system [5]. Most of the efforts put into identifying the contributors to VAP are focused on factors during the intubated period and less so on factors prior to intubation. With efforts made to avoid or delay intubation, there may be underrecognized factors in the pre-intubation period that may increase the risk for VAP.

Non-invasive ventilation/oxygenation (NIVO) can be used in both hypoxemic and hypercapnic respiratory failure for many different pathologies. Different delivery devices make up NIVO and consist of a continuous positive airway pressure (CPAP), bilevel positive airway pressure (BiPAP), and high-flow nasal cannula (HFNC), each having their own indications, benefits, and risks. The rate of NIVO use has been increasing worldwide over the years in an attempt to prevent intubation and IMV [6,7,8,9,10,11]. Recent guidelines consider the use of NIVO to avoid intubation or to prevent re-intubation as an essential practice [12]. The aim of this study was to assess the association between NIVO prior to intubation and the rate of subsequent VAP.

## 2. Materials and Methods

This was a multicenter retrospective cohort study of all adult patients admitted to the medical ICU from three tertiary care academic centers located in three distinct regions (Southeast, Midwest, and Southwest) of the United States. All patients included had to be intubated and mechanically ventilated between 1 January 2019 and 30 December 2021. This study was approved by the Institutional Review Board (21-012140).

An electronic health record was used to collect all data, with the initial patients being identified by all ICU patients who were intubated and mechanically ventilated. We searched these patients to determine if they had a VAP diagnosis by using the ICD-10 code, J95.851. Our primary predictor variable was NIVO type. Other data collected included ICU LOS, hospital LOS, number of days on NIVO before intubation, year of ICU admission, age at ICU admission, sex, race, ethnicity, and medical history, including hypertension, asthma, cancerous tumors, congestive heart failure (CHF), chronic obstructive pulmonary disease COPD, interstitial lung disease (ILD), kidney disease, leukemia, liver disease, lymphoma, peripheral vascular disease, bronchiectasis, solid organ transplant, bone marrow stem cell transplant, and COVID, at the time of admission. These data were collected by reviewing all patients with a VAP diagnosis. If a patient died during admission, their hospital and ICU LOS were not collected, but all other parameters were included.

Ventilator-associated pneumonia was defined as pneumonia that develops in patients receiving IMV 48 h after endotracheal intubation, based on the American Thoracic Society consensus guidelines [13]. To diagnose new pneumonia, patients had to have had clinical changes of fever, increased oxygen requirements, new radiographic change, and lower respiratory tract culture (tracheal aspirate or bronchoalveolar lavage) positive for a pathogen. If these criteria were met, the patient was diagnosed with VAP in the electronic medical record, which allowed for the identification of patients. All patients receiving mechanical ventilation in the ICU of the participating centers received a VAP prevention “bundle”, which included a minimum of 30-degree elevation of the head of the bed, twice daily oral care, and daily interruptions in sedation as appropriate to prevent unnecessarily prolonged intubation. This bundle is standardized across the three included sites due to a shared electronic medical record and shared clinical practice guidelines.

NIVO was defined as the use of CPAP, BiPAP, or HFNC for any duration for respiratory failure (either hypoxic or hypercapnic) during the hospitalization prior to receiving IMV. If only one method of NIVO was used, the patient was included in that respective category. If any combination of NIVO was used, either two or three methods, then they were included in a combination category. If the patient received a continuation of home NIV (either home BiPAP or CPAP), for indications such as sleep apnea, they were included in the no NIVO group. Reasons for NIVO included all causes for acute hypoxic, hypercapnic, or mixed respiratory failure. The specific reason for the use of NIVO or intubation was not collected. The study period included the COVID-19 pandemic.

For those who were diagnosed with VAP, microbiologic organisms detected on respiratory culture (tracheal aspirate or bronchoalveolar lavage) were collected. If a patient had one or more microorganisms of the same Gram-stain category, they were placed in either the Gram-positive or Gram-negative group. If there was a combination of both Gram-positive and Gram-negative microorganisms identified, the patient was placed in the mixed group.

The primary outcome variable was in-hospital VAP association with NIVO. Secondary outcomes consisted of total rate of VAP, in-hospital mortality, ICU LOS, and hospital LOS.

Statistical methods: The sample median and interquartile (IQR) range were used to describe continuous characteristics. The number and percentage of patients were used to describe categorical characteristics. For the primary analysis, we examined the association of NIVO type with in-hospital VAP using single variable logistic regression; odds ratios (ORs) and corresponding 95% confidence intervals (CIs) and *p* values were reported. In the multivariable model, we included all patient demographics and clinical characteristics from Table 1 as covariates. To control the type I error rate at 5%, we applied a Bonferroni correction for our primary analysis, comparing the four NIVO groups to the group who did not have NIVO, after which *p* values < 0.0125 were considered statistically significant. In a post hoc exploratory analysis in the subset of patients who developed VAP, we examined the association of NIVO type with culture staining results (Gram-positive, Gram-negative, and mixed) using Pearson’s chi-squared test. All statistical analyses were conducted using R version 4.1.2 (R Foundation for Statistical Computing, Vienna, Austria).

## 3. Results

A total of 17,302 patients were identified and included in the analysis. The median age was 64 years (IQR 52-72), and 10,694 (61.8%) patients were male. In our cohort, 12,518 (72.4%) patients did not receive NIVO, 1879 (10.9%) patients used CPAP, 690 (4.0%) patients used HFNC, 197 (1.1%) patients used BiPAP, and 2018 (11.7%) patients had a combination of CPAP, HFNC, or BiPAP. Table 1 shows the patients’ demographic information and clinical characteristics overall and according to the NIVO type.

VAP developed in 2.6% (444/17,302) of our total cohort, in 2.3% of patients who did not use NIVO, 1.6% of patients who used CPAP, 2.5% of patients who used HFNC, 8.1% of patients who used BiPAP, and 4.8% of patients who had a combination of NIVO modalities. The percentage of all-cause mortality within the VAP patients was 52.7% (234/444).

In-hospital death occurred in 28.5% (4926/17,302) of our total cohort, in 26.2% of those who did not use NIVO, 24.2% of patients who used CPAP, 35.1% of those who used HFNC, 44.7% of those who used BiPAP, and 43.0% of those who used a combination of NIVO types. The median hospital LOS was 9 days, with a greater LOS in the BiPAP cohort. The median ICU LOS was 3 days, with the longest median LOS noted in the HFNC and combination NIVO cohorts. The median number of days of NIVO was 6, 7, 7, and 8 days in the BiPAP, CPAP, combo NIVO, and HFNC cohorts, respectively. Table 2 shows the occurrence of VAP, death, and other hospital outcomes according to the NIVO type.

The associations of NIVO modality with VAP are shown in Table 3. The odds of developing VAP were 3.11 times higher among patients who used only BiPAP compared to those who did not receive NIVO after adjusting for patient demographics, illness severity scoring, and comorbidities (95% CI, 1.80–5.37, *p* <0.001). The odds of VAP were also higher among those who had a combination of NIVO types compared to those who did not have NIVO (adjusted OR = 1.91, 95% CI 1.49–2.44, *p* < 0.001). CPAP and HFNC did not show any association with VAP when compared to no NIVO in our cohort.

In an exploratory analysis, we examined the association of the NIVO type with the Gram-staining (Gram-positive or Gram-negative) results in the subset of patients who developed VAP. We did not find evidence of an association of the NIVO type with the Gram-staining results (*p* = 0.38, Table A1).

## 4. Discussion

In adult medical ICU patients who required intubation and IMV, the use of BiPAP prior to intubation was associated with significantly higher odds of developing VAP.

Aside from its high mortality rate, VAP also increases the length of IMV by up to 11.5 days and increases the cost of a patient’s hospitalization by approximately USD 47,000 (USD 21,890–72,578) [1,14]. The mean incidence of VAP is about 2.8%, or 1–2.5 cases per 1000 ventilator days in North America specifically [3,15]. In an attempt to reduce VAP and its associated worse outcomes, prevention bundles are often used in ICUs and include oral care with toothbrushing, minimizing sedation, minimizing the days of IMV, avoiding physical deconditioning while on IMV, and head of the bed elevation to prevent the reflux of gastric secretions, but the use of these prevention methods have mixed results [16,17]. Further efforts have focused on other modifiable factors, such as investigating alternative endotracheal tube materials or even antimicrobial coatings, subglottic secretion suctioning, and selective oral and gastric decontamination, but have even less evidence in preventing VAP and improving outcomes [3,18]. Other hypothesized factors for increased VAP risk include bacterial colonization due to micro-aspiration and defective mucociliary clearance [3]. Specific diseases, such as COPD, have been as identified as independent risk factors for VAP secondary to microbacterial colonization from micro-aspiration [15]. In COPD, the severities of hyperinflation and dyspnea have also been identified as independent risk factors for gastroesophageal reflux [15]. The relationship between NIVO and micro-aspiration is unclear, but it is possible that the presence of positive pressure with gastroesophageal reflux may contribute to bacterial passage into the airway. In cases of hypoventilation and elevated carbon dioxide, there may also be a decreased level of consciousness, increasing the risk for aspiration while using NIVO. Our study did not identify an association between VAP following specific NIVO modalities and Gram-staining results or resistant bacteria. The most frequently isolated bacteria were methicillin-susceptible staphylococcus aureus (31.8%), klebsiella (18.9%), and pseudomonas (18.7%). These are less commonly found in the GI tract, so one may argue against micro-aspiration and lean more towards other causes of VAP. Typically, the microorganisms that are responsible for VAP are influenced by geographic area, but these findings appear to be consistent with the commonly reported bacteria in VAP [19].

The practice that has been found to most consistently improve VAP outcomes is a strategy of avoiding intubation, minimizing IMV duration, and early mobilization [3,20,21,22,23]. NIVO has been shown to be a method to help avoid intubation and IMV in respiratory failure [3,7,24,25,26,27]. NIVO includes the use of BiPAP, CPAP, or HFNC, while NIV typically includes just BiPAP or CPAP. NIVO provides benefit from the delivery of an increased fraction of inspired oxygen and/or increased flow rates, allowing for a decrease in the work of breathing, improved alveolar recruitment, and improved ventilation [28]. During NIVO, patients spontaneously breathe through either a closed or open system with humidification, and programmed amounts of pressure and oxygen are provided to the patient. The use of NIVO has expanded from acute hypercapnic respiratory failure to acute hypoxemic respiratory failure, allowing for more opportunities for clinical application. In acute hypercapnic respiratory failure, the use of NIV has been shown to reduce mortality and the need for IMV [29,30]. The use of NIV in hypoxemic respiratory failure has shown a reduction in the need for endotracheal intubation, but not mortality [31]. The use of NIV for hypercapnic respiratory failure fails in around 12.8–32.5% of patients, with failure defined as death or the need for IMV [32]. NIVO can fail for both delivery system reasons, such as patient intolerance or dyssynchrony, as well as ventilatory failure, such as a lack of response to therapy [29]. The early identification of failure is key, as patients with late failure requiring mechanical ventilation may have a less favorable outcome including increased hospital mortality, which was noted in one study to be 41.5% versus 16.1% without NIV failure, and an increased hospital LOS [29,33]. Some indications for failure include higher clinical severity scores (e.g., DECAF score–dyspnea, eosinopenia, consolidation, acidemia, and atrial fibrillation), an increased age, and the presence of acute respiratory distress syndrome (ARDS) [34]. The use of HFNC in acute hypoxic respiratory failure was previously shown to have improved 90-day mortality, but the need for IMV compared to standard oxygen or NIV was not significantly different [35]. Overall, this indicates that NIVO can be an appropriate therapy option in both hypoxic and hypercapnic respiratory failure. However, with the knowledge that NIVO can still fail, requiring escalation to IMV, and that a delay in escalation may worsen outcomes, the success and tolerance of NIVO should be closely monitored, and IMV should be initiated without significant delay. In our study, the utilization of a combination of NIVO methods may signal an intolerance or poor response to an initial NIVO method and subsequent failure that was not properly recognized.

Of the three commonly used NIVO methods, our study found that the use of BiPAP or a combination of NIVO types prior to IMV increased the risk of VAP. BiPAP differs from CPAP by the application of two levels of pressure, with a set inspiratory pressure, which is higher than a set expiratory pressure. Respiratory failure, particularly hypoxemic respiratory failure, can result in a high respiratory drive [36,37]. For patients with a high respiratory drive, controlling the inspired tidal volume on NIVO is challenging and often larger than the ideal protective ventilation volume, with an average tidal volume being around 10 mL/kg (goal 6–8 mL/kg), placing patients at risk of self-induced lung injury [36,38]. In a similar way to ventilator-associated lung injury, large tidal volumes can cause excessive lung strain, defined as the ratio between end-expiratory lung volume and tidal volume, stretching the lung beyond a physiologic limit and causing tissue injury [37]. The resultant tissue damage places the lungs at a higher risk of superimposed infection [39]. Assessments of the airway pressure, such as the peak and plateau pressures, on BiPAP are unreliable as it is not a perfectly closed system. This means that further measures to decrease lung injury, including reducing the plateau pressure and driving pressure, cannot be controlled. The driving pressure is calculated as the difference between the plateau pressure and end expiratory airway pressure [40]. The driving pressure uses the rationale of tailoring the tidal volume to the respiratory system compliance, often dictated by the number of functioning alveoli, and should therefore correlate better with functional lungs other than tidal volume [40]. In trials of ARDS patients receiving IMV, the driving pressure is most strongly associated with an improved survival, with part of the rationale being that a decreased driving pressure decreases the lung stretch and associated injury [41]. It is possible that the limited control of variables, such as the driving pressure and tidal volume, which is most likely more pronounced when receiving BiPAP due to the addition of an increased inspiratory pressure, results in tissue damage and a high susceptibility to infection when receiving IMV.

Despite the increased incidence of VAP with BiPAP or combination NIVO, our study noted a similar overall incidence of VAP (2.6%) compared to previously reported rates [15]. Across all groups, the ICU LOS was comparable; however, the total hospital LOS was the highest in the BiPAP group. Hospital mortality was also the highest in the BiPAP (44.7%) and combination NIVO (43.0%) groups. It may be that, with the higher hospital mortality in the BiPAP group, the overall BiPAP group ICU LOS may be underestimated.

Our study has limitations. Firstly, it is a retrospective analysis, but it allows for the temporal analysis of the relationship between utilizing NIVO and VAP occurrence. Further, data regarding the specific type of mask interfaces or pressure settings used while a patient was on NIV were not available. As discussed previously, studies have already shown that the settings on CPAP and BiPAP are unreliable secondary to additional patient effort [36,38]. Similarly, parameters on IMV were not able to be collected including the driving pressure, compliance, and tidal volume, which can be important factors in the development of VAP. Finally, the specific indications for the use of NIVO or IMV were not able to be collected. As previously discussed, some diseases, such as COPD, have an independent relationship with the development of VAP [15]. The analysis was adjusted for patient comorbidities but it cannot account for whether the patients had a hypoxemic or hypercapnic respiratory failure.

## 5. Conclusions

The use of BiPAP, or combination NIVO, significantly increases the odds of developing VAP once receiving IMV. The reasons for this may include the limited control of pressure and volume while receiving NIVO, or micro-aspiration. This does not suggest that NIVO should not be used to limit or prevent IMV, but that certain modalities may eventually increase the odds of developing VAP. In addition, patients receiving NIVO should be monitored closely for signs of NIVO failure or intolerance with escalation to IMV when appropriate. A further study may be required to understand the effect of BiPAP in this patient population.

## Figures and Tables

**Table 1:** Patient demographic information and clinical characteristics according to non-invasive ventilator type.

	No NIVO (N = 12,518)	CPAP (N = 1879)	HFNC (N = 690)	BIPAP (N = 197)	Combo (N = 2018)	Total (N = 17,302)
Age at ICU admission	63 (51, 72)	66 (57, 74)	62 (50, 70)	65 (54, 72)	65 (55, 73)	64 (52, 72)
Sex (male)	7778 (62.1)	1241 (66.1)	414 (60.0)	113 (57.4)	1148 (56.9)	10,694 (61.8)
Race						
White	10,710 (85.6)	1672 (89.0)	589 (85.4)	173 (87.8)	1795 (88.9)	14,939 (86.3)
African American	743 (5.9)	90 (4.8)	50 (7.2)	9 (4.6)	75 (3.7)	967 (5.6)
Unknown	207 (1.7)	23 (1.2)	10 (1.4)	2 (1.0)	36 (1.8)	278 (1.6)
Choose not to disclose	105 (0.8)	11 (0.6)	6 (0.9)	0 (0.0)	13 (0.6)	135 (0.8)
Other	753 (6.0)	83 (4.4)	35 (5.1)	13 (6.6)	99 (4.9)	983 (5.7)
Ethnicity						
Hispanic or Latino	338 (2.7)	34 (1.8)	28 (4.1)	6 (3.0)	47 (2.3)	453 (2.6)
Not Hispanic or Latino	11,537 (92.2)	1770 (94.2)	620 (89.9)	180 (91.4)	1870 (92.7)	15,977 (92.3)
Unknown	265 (2.1)	36 (1.9)	16 (2.3)	5 (2.5)	45 (2.2)	367 (2.1)
Choose not to disclose	160 (1.3)	19 (1.0)	12 (1.7)	2 (1.0)	26 (1.3)	219 (1.3)
Unable to provide	47 (0.4)	4 (0.2)	2 (0.3)	0 (0.0)	5 (0.2)	58 (0.3)
Other	171 (1.4)	16 (0.9)	12 (1.7)	4 (2.0)	25 (1.2)	228 (1.3)
Hypertension	7014 (56.0)	1319 (70.2)	364 (52.8)	127 (64.5)	1279 (63.4)	10,103 (58.4)
Asthma	1199 (9.6)	254 (13.5)	63 (9.1)	32 (16.2)	294 (14.6)	1842 (10.6)
Solid organ malignancy	2570 (20.5)	363 (19.3)	129 (18.7)	48 (24.4)	394 (19.5)	3504 (20.3)
CHF	2473 (19.8)	559 (29.7)	166 (24.1)	67 (34.0)	652 (32.3)	3917 (22.6)
COPD	1431 (11.4)	341 (18.1)	120 (17.4)	50 (25.4)	540 (26.8)	2482 (14.3)
ILD	302 (2.4)	59 (3.1)	63 (9.1)	10 (5.1)	106 (5.3)	540 (3.1)
CKD	3221 (25.7)	609 (32.4)	154 (22.3)	67 (34.0)	628 (31.1)	4679 (27.0)
Hematologic malignancy	201 (1.6)	42 (2.2)	17 (2.5)	9 (4.6)	59 (2.9)	328 (1.9)
Liver disease	1331 (10.6)	132 (7.0)	59 (8.6)	20 (10.2)	162 (8.0)	1704 (9.8)
Lymphoma	376 (3.0)	50 (2.7)	27 (3.9)	24 (12.2)	116 (5.7)	593 (3.4)
PVD	858 (6.9)	150 (8.0)	49 (7.1)	28 (14.2)	203 (10.1)	1288 (7.4)
Bronchiectasis	197 (1.6)	36 (1.9)	30 (4.3)	5 (2.5)	61 (3.0)	329 (1.9)
Solid organ transplant	1432 (11.4)	168 (8.9)	141 (20.4)	17 (8.6)	157 (7.8)	1915 (11.1)
Bone marrow/stem cell transplant	3079 (24.6)	473 (25.2)	182 (26.4)	79 (40.1)	604 (29.9)	4417 (25.5)
COVID-19 at admission	196 (1.6)	32 (1.7)	51 (7.4)	5 (2.5)	98 (4.9)	382 (2.2)
SOFA score at ICU admission	9 (6, 12)	10 (6.5, 13.5)	10 (7, 13)	10 (7, 13)	8 (4, 12)	9 (6, 12)
APACHE 3 score at ICU admission	101 (78, 124)	104 (785, 129.5)	103 (80, 126)	98.5 (73, 124)	105 (81.5, 128.5)	102 (79, 125)

Abbreviations: NIVO, non-invasive ventilation or oxygenation; ICU, intensive care unit; CHF, congestive heart failure; COPD, chronic obstructive pulmonary disease; ILD, interstitial lung disease; PVD, peripheral vascular disease; CKD, chronic kidney disease; SOFA, sequential organ failure assessment; APACHE 3, acute physiology and chronic health evaluation. Sample median (25th percentile and 75th percentile) is shown for continuous characteristics. Number of patients (column percentage) is shown for categorical characteristics.

**Table 2:** Ventilator-associated pneumonia and other hospital-related outcomes according to NIVO type.

	No NIVO (N = 12,518)	CPAP (N = 1879)	HFNC (N = 690)	BIPAP (N = 197)	Combo (N = 2018)	Total (N = 17,302)
VAP	285 (2.3)	30 (1.6)	17 (2.5)	16 (8.1)	96 (4.8)	444 (2.6)
ICU length of stay, days	2 (1, 4)	3 (2, 6)	6 (3, 11)	5 (2, 11)	6 (3, 11)	3 (1, 6)
Length of NIVO, days	N/A	7 (1, 17)	8 (1, 17)	6 (1, 13)	7 (1, 17)	7 (1, 17) *
Hospital LOS, days	8 (5, 15)	10 (6, 18)	16 (9, 32)	20 (9, 42)	15 (9, 27)	9 (5, 18)
KM Hospital LOS **	8 (5, 18)	10 (7, 20)	20 (10, 41)	28 (9, 58)	17 (10, 33)	10 (6, 21)
Death	3274 (26.2)	455 (24.2)	242 (35.1)	88 (44.7)	867 (43.0)	4926 (28.5)

Abbreviations: VAP, ventilator-associated pneumonia; NIVO, non-invasive ventilation or oxygenation; ICU, intensive care unit; LOS, length of stay; KM, Kaplan–Meier. Sample median (25th percentile and 75th percentile) is shown for continuous characteristics. Number of patients (column percentage) is shown for categorical characteristics. * In the subset of patients who had NIVO. ** The reverse Kaplan–Meier method was used to estimate the length of stay where patients who died in the hospital were censored on the death date.

**Table 3:** Logistic regression analysis evaluating the association of non-invasive ventilator type with ventilator-associated pneumonia among intubated patients who were admitted to the intensive care unit.

	Association with in-Hospital VAP			
	Unadjusted Analysis		Multivariable Analysis	
NIVO Type	OR (95% CI)	*p*-Value	OR (95% CI)	*p*-Value
None	Reference	N/A	Reference	N/A
CPAP	0.70 (0.48, 1.02)	0.062	0.72 (0.49, 1.05)	0.091
HFNC	1.08 (0.66, 1.78)	0.75	0.97 (0.58, 1.60)	0.89
BiPAP	3.79 (2.25, 6.41)	<0.001	3.11 (1.80, 5.37)	<0.001
Combo	2.14 (1.69, 2.72)	<0.001	1.91 (1.49, 2.44)	<0.001

Odds ratios (ORs), 95% confidence intervals (CIs), and *p* values were estimated from single variable (unadjusted) and multivariable (adjusted) logistic regression models with in-hospital VAP as the outcome variable. Multivariable logistic regression models were adjusted for age at ICU admission, sex, race, ethnicity, and all of the medical history variables in Table 1.

## Data Availability

The data are available upon reasonable requests.

## Appendix A

**Table A1:** Exploratory examination of the association of NIV type with staining results in the subset of patients who developed VAP. Numbers may add up to more than 100% because some patients had multiple bacteria isolated during their ventilator-associated pneumonia.

NIVO Type							
	None	CPAP	HFNC	BIPAP	Combo	Total	*p*-Value
	(N = 285)	(N = 30)	(N = 17)	(N = 16)	(N = 96)	(N = 444)	
Staining result							
Gram-positive	193 (67.7)	21 (70.0)	7 (41.2)	13 (81.3)	60 (62.5)	294 (66.2)	0.38
Gram-negative	78 (27.4)	7 (23.3)	9 (52.9)	2 (12.5)	30 (31.3)	126 (28.4)	
Mixed	14 (4.9)	2 (6.7)	1 (5.9)	1 (6.3)	6 (6.3)	24 (5.4)	
Bacteria type(s)							
Achromobacter	11 (3.9)	1 (3.3)	1 (5.9)	0	3 (0.3)	16 (3.6)	
Acinetobacter	2 (0.7)	0	0	2 (12.5)	0	4 (0.9)	
Burkholderia	4 (1.4)	0	1 (5.9)	1 (6.3)	1 (0.1)	7 (1.6)	
Citrobacter	15 (5.3)	3 (10)	0	1 (6.3)	4 (0.4)	23 (5.2)	
Corynebacterium	1 (0.4)	0	0	0	0	1 (0.2)	
Escherichia coli	28 (9.8)	4 (13.3)	0	1 (6.3)	11 (1.1)	44 (9.9)	
Elizabethkingia	0	0	1 (5.9)	0	0	1 (0.2)	
Enterobacter	12 (4.2)	3 (10)	0	0	5 (0.5)	20 (4.5)	
Haemophilus	5 (1.8)	1 (3.3)	0	1 (6.3)	1 (0.1)	8 (1.8)	
Klebsiella	54 (18.9)	5 (16.7)	2 (11.8)	3 (18.8)	20 (2.1)	84 (18.9)	
Moraxella	9 (3.2)	1 (3.3)	0	0	1 (0.1)	11 (2.5)	
Morganella	2 (0.7)	0	1 (5.9)	0	1 (0.1)	4 (0.9)	
MRSA	3 (1.1)	0	0	0	3 (0.3)	6 (1.4)	
MSSA	87 (30.5)	9 (30)	10 (58.8)	2 (12.5)	33 (3.4)	141 (31.8)	
Proteus	0	1 (3.3)	0	0	0	1 (0.2)	
Pseudomonas	56 (19.6)	5 (16.7)	3 (17.6)	5 (31.3)	14 (1.4)	83 (18.7)	
Serratia	32 (11.2)	2 (6.7)	2 (11.8)	1 (6.3)	9 (0.9)	46 (10.4)	
Stenotrophomonas	10 (3.5)	1 (3.3)	0	2 (12.5)	9 (0.9)	22 (5)	
Streptococcus pneumoniae	2 (0.7)	0	0	0	2 (0.2)	4 (0.9)	
Streptococcus agalactiae	3 (1.1)	1 (3.3)	0	0	3 (0.3)	7 (1.6)	

Number of patients (column percentage) is shown for each NIVO type. *p*-value from chi-squared test. MRSA—methicillin-resistant staphylococcus aureus. MSSA—methicillin-susceptible staphylococcus aureus.
